# Domesticated equine species and their derived hybrids differ in their fecal microbiota

**DOI:** 10.1186/s42523-020-00027-7

**Published:** 2020-03-16

**Authors:** J. E. Edwards, A. Schennink, F. Burden, S. Long, D. A. van Doorn, W. F. Pellikaan, J. Dijkstra, E. Saccenti, H. Smidt

**Affiliations:** 1 Laboratory of Microbiology, Wageningen University & Research, 6708 WE Wageningen, Netherlands; 2 Present address: Micreos Human Health B.V, Bilthoven, Netherlands; 3 The Donkey Sanctuary, Sidmouth, Devon EX10 ONU UK; 4 Division of Nutrition, Department of Farm Animal Health, Faculty of Veterinary Medicine, Utrecht University, 3584 CM Utrecht, Netherlands; 5 Department of Equine Health, Faculty of Veterinary Medicine, Utrecht University, 3584 CL Utrecht, Netherlands; 6 Animal Nutrition Group, Wageningen University & Research, 6708 WD Wageningen, Netherlands; 7 Laboratory of Systems and Synthetic Biology, Wageningen University & Research, Wageningen, the Netherlands

**Keywords:** Feces, Barcoded amplicon sequencing, Bacteria, Archaea, Anaerobic fungi, Pony, Donkey, Mule, Hinny

## Abstract

**Background:**

Compared to horses and ponies, donkeys have increased degradation of dietary fiber. The longer total mean retention time of feed in the donkey gut has been proposed to be the basis of this, because of the increased time available for feed to be acted upon by enzymes and the gut microbiota. However, differences in terms of microbial concentrations and/or community composition in the hindgut may also underpin the increased degradation of fiber in donkeys. Therefore, a study was conducted to assess if differences existed between the fecal microbiota of pony, donkey and hybrids derived from them (i.e. pony × donkey) when fed the same forage diet.

**Results:**

Fecal community composition of prokaryotes and anaerobic fungi significantly differed between equine types. The relative abundance of two bacterial genera was significantly higher in donkey compared to both pony and pony x donkey: *Lachnoclostridium* 10 and ‘probable genus 10’ from the *Lachnospiraceae* family. The relative abundance of *Piromyces* was significantly lower in donkey compared to pony × donkey, with pony not significantly differing from either of the other equine types. In contrast, the uncultivated genus SK3 was only found in donkey (4 of the 8 animals). The number of anaerobic fungal OTUs was also significantly higher in donkey than in the other two equine types, with no significant differences found between pony and pony × donkey. Equine types did not significantly differ with respect to prokaryotic alpha diversity, fecal dry matter content or fecal concentrations of bacteria, archaea and anaerobic fungi.

**Conclusions:**

Donkey fecal microbiota differed from that of both pony and pony × donkey. These differences related to a higher relative abundance and diversity of taxa with known, or speculated, roles in plant material degradation. These findings are consistent with the previously reported increased fiber degradation in donkeys compared to ponies, and suggest that the hindgut microbiota plays a role. This offers novel opportunities for pony and pony × donkey to extract more energy from dietary fiber via microbial mediated strategies. This could potentially decrease the need for energy dense feeds which are a risk factor for gut-mediated disease.

## Background

Hindgut microbial fermentation of plant material in equines results in the generation of volatile fatty acids, which are a major source of energy for the equine host [1–3]. The improvement of fiber utilization would decrease the need for energy dense concentrate feeds, which have been linked to increased risk of colic and adverse effects on the hindgut microbiota [4, 5]. Furthermore, in regions where only limited feed sources and poor quality forage are available, the efficient use of available fibrous feed sources by the animal is important to be able to meet its energy requirements. Optimizing fiber utilization can, therefore, have a beneficial impact on the health and welfare of equines.

The world’s 116 million domesticated equines includes horses/ponies (52%), donkeys (40%) and mules/hinnies (8%) [6]. Domesticated donkeys have evolved from ancestors that inhabited semi-arid and often mountainous environments which had sparse vegetation [7]. This is in contrast to the grassland plains where horses evolved. As such, it is not surprising that as part of their evolution donkeys developed an improved ability to degrade fiber which enables them to survive better on lignin rich, low energy, fibrous plants [7].

Compared to horses/ponies, donkeys have increased dietary fiber digestibility and decreased feed intake [8, 9]. This is true regardless of dietary fiber content [9], although differences in dry matter (DM) digestion between species become more pronounced when diet quality decreases [10]. The increased mean retention time (MRT) of feed particles in the donkey gut, compared to horses/ponies [8, 9], has been suggested to be the cause of this increased fiber digestibility. It has been hypothesized that this is due to the increased time available for the dietary material to be acted upon by enzymes and the gut microbiota [8]. However, it is also possible that different host species select for/harbor different gut microbial communities, as has been previously shown in a study with two species of deer and a hybrid derived from them [11].

The equine hindgut microbiota is comprised of bacteria, anaerobic fungi, archaea, protozoa, and viruses. Of these, mainly bacteria have been studied to date [12]. Anaerobic fungi and certain bacteria are the only hindgut microbes directly involved in fiber degradation. Archaea and viruses are unable to degrade dietary material, and protozoa make a limited contribution to fiber degradation in the horse hindgut [13]. It has been previously reported that the total number of anaerobic bacteria and cellulolytic bacteria in the equine caecum does not significantly differ between ponies and donkeys fed the same diet [14]. However, a donkey anaerobic fungal isolate of *Piromyces citronii* was found to be superior to that of a pony isolate of the same species in terms of degrading cellulose [15]. Anaerobic fungi are powerful fiber degraders due to their combined invasive growth and broad array of highly effective plant degrading enzymes [16, 17]. In ruminants fed poor quality herbage or straw anaerobic fungi are known to increase feed dry matter digestibility by 7–9% [18], however, their contribution to feed degradation in the equine hindgut remains to be determined.

Whilst numerous studies have explored the composition of the bacterial community in the hindgut of horses and ponies [5, 12, 19–31], only limited studies have been performed on other equine species to date. Liu et al. [32] described the fecal microbiota of domesticated donkeys, and noted differences in the relative abundances of bacterial phyla compared to those previously reported in horses and ponies. The fecal microbiota of the Tibetan wild ass has also been studied [33], which is an equine species that is more closely related to the domesticated donkey than horse [34]. Captive Tibetan wild ass was reported to have decreased prokaryotic alpha diversity and a different community composition relative to wild Tibetan wild ass, and authors speculated that this difference was mainly diet related [33]. No studies to date have been performed on mules or hinnies despite their global population of 9.6 million [6], the majority of which are purpose bred working animals.

In this study, fecal samples from domesticated ponies (*Equus caballus*), donkeys (*Equus africanus asinus*) and their derived hybrids (i.e. pony × donkey) fed the same forage diet were characterized in order to assess if the microbiota present in the equine hindgut differs between domesticated equine species and their derived hybrids. The fecal prokaryotic and anaerobic fungal community composition was determined using barcoded amplicon sequencing, and microbial concentrations measured using quantitative PCR. It was hypothesized that the fecal microbiota of donkey differed from that of pony in relation to taxa involved in fiber degradation, with the hybrid being intermediate relative to donkey and pony.

## Results

The animals used in this study were all healthy adults that had no known history of any gut-mediated disease. The animals included ponies (*n* = 8), donkeys (*n* = 8) and mules/hinnies (*n* = 8), and each equine type was composed of three females and five males. As the parentage of the mules/hinnies was not known, they are referred to as pony x donkey in this study. Further details of the individual animals are given in Additional file 1: Table S1.

### Fecal dry Matter Content & Microbial Concentrations

The percentage fecal DM content (average ± SD) of pony (18.2 ± 2.0), pony × donkey (19.0 ± 1.7) and donkey (20.3 ± 1.5) did not significantly differ between equine types (*P* = 0.07). Equine types were also not significantly different with respect to fecal concentrations of bacteria, archaea or anaerobic fungi when expressed on a fresh weight basis (Table 1). When analyzed on a DM basis, equine types also did not differ in fecal concentrations of bacteria (*P* = 0.54), archaea (*P* = 0.70) and anaerobic fungi (*P* = 0.14) (data not shown).

**Table 1:** Effect of equine type on fecal microbial concentrations

Microbe^a^	Donkey	Pony	Pony × Donkey	*P* value
Bacteria	52.4 ± 16.8	51.0 ± 11.9	59.7 ± 21.4	0.70
Archaea	2.27 ± 0.34	1.85 ± 0.75	2.08 ± 1.04	0.39
Anaerobic Fungi	1.17 ± 0.50	2.05 ± 1.22	1.78 ± 0.82	0.25

^a^Average values (*n* = 8) ± standard deviation are given for 16S (bacteria and archaea) and 5.8S (anaerobic fungi) rRNA gene copies expressed × 10^8^ gene copies per g fresh weight of feces

### Prokaryotic community composition

Bacteria (97.1 ± 1.14% of the 16S rRNA gene sequences) were represented by 1203 different operational taxonomic units (OTUs). Of these, 319 OTUs were detected at least once in all three equine types, and 18 OTUs were detected in all animals. The 1203 bacterial OTUs could be summarized to 172 different genus-level phylogenetic groupings. The archaea (2.9 ± 1.14% of the 16S rRNA gene sequences) were represented by ten OTUs. Of these, four OTUs were detected at least once in all three equine types and one OTU was detected in all animals. The ten OTUs could be summarized to two different genus-level phylogenetic groupings. Of the 17 phyla detected in total, the following six were predominant (> 1%): *Firmicutes*, *Bacteroidetes*, *Verrucomicrobia*, *Fibrobacteres, Spirochaetes* and *Euryarchaeota* (Additional file 2: Figure S1).

We did not observe a significant difference between equine types (*P* = 0.43) with respect to the number of OTUs observed in pony (average ± SD; 265 ± 9.9), donkey (258 ± 12.0) and pony × donkey (267 ± 18.7). Similarly, the ‘Phylogenetic Diversity’ metric used to assess alpha diversity was also not significantly different between equine types (*P* = 0.30): pony (average ± SD; 17.5 ± 0.44), donkey (17.8 ± 0.30) and pony × donkey (17.5 ± 0.63).

**Fig. 1:**
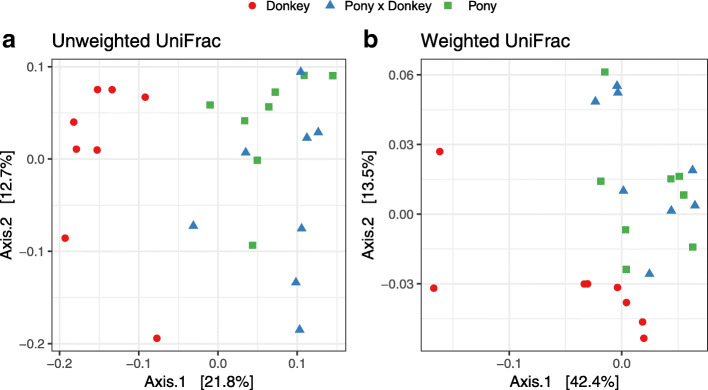
Unweighted (**a**) and weighted (**b**) UniFrac based principal co-ordinates analysis of the fecal prokaryotic community composition of the different equine types at the OTU level. Analysis used Log_10_ transformed data, and the percentage values given on each axis indicate the amount of total variation represented

In terms of beta diversity at the OTU level, the prokaryotic community composition in donkey separated from both pony and pony × donkey along the first axis in the unweighted UniFrac principal co-ordinate analysis (PCoA), whilst pony and pony × donkey did not differ (Fig. 1a). Using weighted UniFrac PCoA, the separation of donkey from pony and pony × donkey did not occur along one axis, instead separating along a diagonal line (Fig. 1b).

**Fig. 2:**
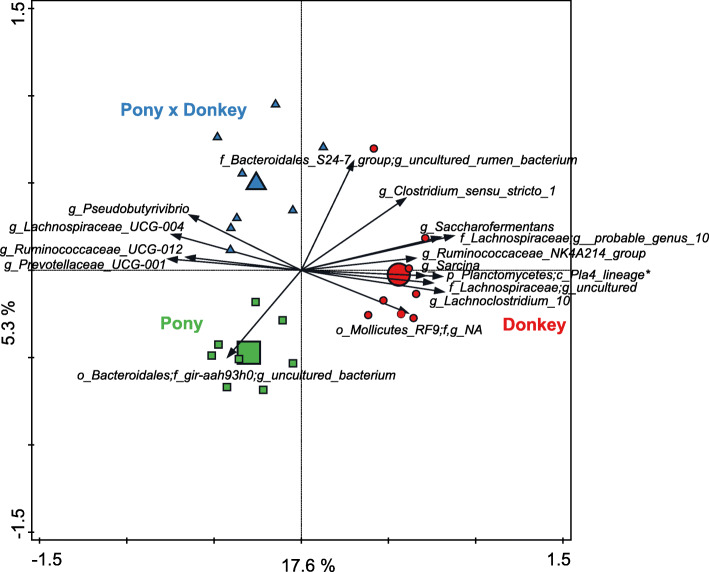
Redundancy analysis triplot showing the relationship between the top 15 prokaryotic genus-level phylogenetic groupings of the OTUs for which the variation is best explained by the constrained axes. Arrow length indicates the variance that can be explained by equine type, with the perpendicular distance of the equine types to the arrow indicating the relative abundance of the genus-level phylogenetic grouping. Arrow labels indicate the taxonomic affiliation of genus-level phylogenetic groups, with the level (i.e. phylum (p), class (c), order (o), family (f) or genus (g)) and taxon (as defined by the Silva 16S rRNA database) that the groups could be reliably assigned to. For example “g__*Pseudobutyrivibrio”* represents an OTU reliably assigned to the genus *Pseudobutyrivibrio*, whereas “o_*Mollicutes*_RF9;f,g__NA” was reliably assigned to the order *Mollicutes*_RF9 but the family and genus could not be annotated (NA). *Due to space constraints, one of the arrow labels was shortened on the plot and the full label is as follows: p_*Planctomycetes*;c__Pla4_lineage;o__uncultured_rumen_bacterium;f,g__NA. Equine type means (large symbols) and individual samples (small symbols) are coded by equine type: donkey (red circle), pony (green square) and pony × donkey (blue triangle). Equine type explained 22.8% of the total variation in the dataset, and the plot axes are labelled with the amount of variation they represent

Redundancy analysis (RDA) using genus level groupings of the OTUs indicated that equine type significantly contributed to explaining the observed variation in fecal prokaryotic community composition (*P* < 0.01), accounting for 22.8% of the overall variation in the dataset. Donkey separated from both pony and pony × donkey along the horizontal canonical axis, which represented 17.6% of the variation in prokaryotic community composition (Fig. 2). Pony and pony × donkey separated from each other along the second canonical axis, which represented 5.3% of the variation in the prokaryotic composition data. Five genus level phylogenetic groupings were strongly positively associated with donkey. Three of these could be annotated to the genus level (*Sarcina*, *Ruminococcaceae* NK4A214 group and *Lachnoclostridium* 10), whilst the other two could only be annotated to the family (*Lachnospiraceae*) or order (‘uncultured rumen bacterium’ in the Pla4 lineage of the phylum *Planctomycetes*) level. One genus level phylogenetic grouping was clearly positively associated with pony, and was annotated as an uncultured bacterium belonging to the family ‘gir-aah93h0’ within the order *Bacteroidales*. No genus level phylogenetic grouping was positively associated with pony × donkey.

A Kruskal-Wallis test indicated that the relative abundance of two of the genus level groups significantly different between equine types: the genus *Lachnoclostridium* 10 (*P* = 0.03) and an uncultured genus called ‘probable genus 10’ from the *Lachnospiraceae* family (*P* = 0.01). Dunn’s Sidak post-hoc analysis showed that the relative abundance of *Lachnoclostridium* 10 was significantly higher in donkey (average percentage relative abundance ± SD, 1.43 ± 0.91) compared to both pony × donkey (0.05 ± 0.11, *P* < 0.001) and pony (0.09 ± 0.14, *P* < 0.01). We did not observe a significant difference between pony × donkey and pony (*P* = 0.98). *Lachnoclostridium* 10 was detected in all eight of the donkeys sampled, whereas for pony × donkey and pony it was detected in only two and three animals, respectively, at lower relative abundances compared to donkey. With ‘probable genus 10’, Dunn’s Sidak post-hoc analysis also showed that the relative abundance of this genus was significantly higher in donkey (0.58 ± 0.19) compared to pony × donkey (0.07 ± 0.12, *P* < 0.01) and pony (not detected, *P* < 0.0001). We did not observe a significant difference between pony × donkey and pony (*P* = 0.73). This genus was detected in all eight donkeys, whereas in the pony × donkey it was detected in only three of the animals at lower relative abundances compared to donkey.

### Anaerobic fungal community composition

**Fig. 3:**
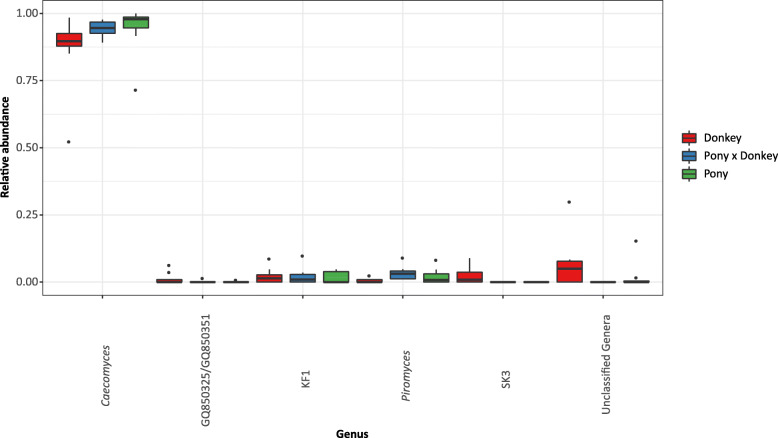
Boxplot showing the anaerobic fungal genera detected in the different equine types. Genera that could not be classified are grouped as ‘Unclassified Genera’. Boxes show the 25th and 75th percentiles with the median represented by a horizontal line. Whiskers show the data range with the exception of any outliers, which are indicated as data points

In the anaerobic fungal sequence data 72 OTUs were detected. Of these, 13 OTUs were detected at least once in all three equine types, and three OTUs were detected in all animals. The 72 OTUs could be summarized to five different genus level groups. The genus *Caecomyces* was most predominant in the anaerobic fungal community of all of the animals (Fig. 3). The number of OTUs observed was significantly different between equine types (*P* < 0.01), with donkeys (average ± SD; 17 ± 5.2) having more OTUs than both pony (10 ± 4.6) and pony × donkey (10 ± 2.97). However, the ‘Phylogenetic Diversity’ metric used to assess alpha diversity did not significantly differ between equine types (*P* = 0.27): donkey (average ± SD; 0.40 ± 0.186), pony (0.28 ± 0.177) and pony × donkey (0.31 ± 0.072).

**Fig. 4:**
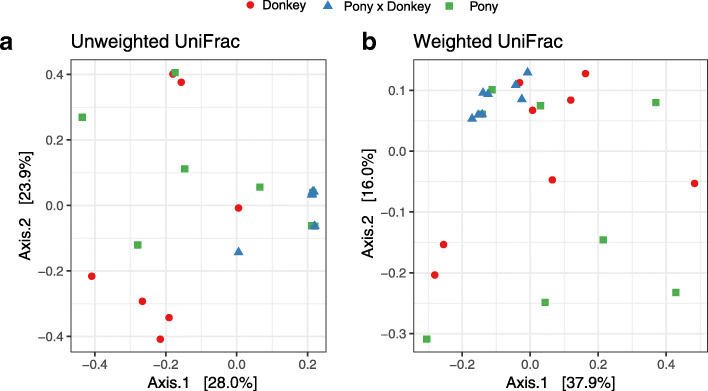
Unweighted (**a**) and weighted (**b**) UniFrac based principal co-ordinates analysis of the fecal anaerobic fungal community composition of the different equine types at the OTU level. Analysis used Log_10_ transformed data, and the percentages values labelled on each axis indicate the amount of total variation represented

In terms of beta diversity at the OTU level, weighted and unweighted UniFrac PCoA showed that pony × donkey anaerobic fungal community composition varied less between individuals than that of donkey and pony (Fig. 4). Separation of pony × donkey from donkey occurred along the first axis of the unweighted PCoA, but no separation of these two equine types from pony was seen (Fig. 4a). No obvious separation of the samples by equine type was observed in the weighted PCoA (Fig. 4b), presumably due to the predominance of *Caecomyces* in all animals.

**Fig. 5:**
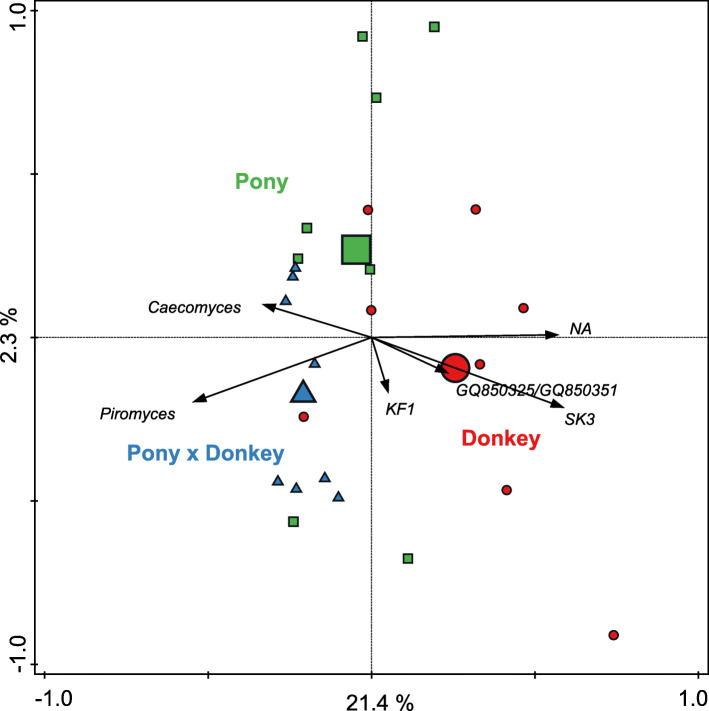
Redundancy analysis triplot showing the relationship between the anaerobic fungal genus-level phylogenetic groupings of the OTUs for which the variation is best explained by the constrained axes. Arrow length indicates the variance that can be explained by equine type, with the perpendicular distance of the equine types to the arrow indicating the relative abundance of the genus-level phylogenetic grouping. Arrow labels indicate the taxonomic affiliation that the genera could be reliably assigned to. For example, ‘g_SK3’ represents a grouping reliably assigned to the SK3 genus, whereas ‘NA’ indicates that it was reliably assigned to the family *Neocallimastigaceae* but the genus could not be annotated. Equine type means (large symbols) and individual samples (small symbols) are coded by equine type: donkey (red circle), pony (green square) and pony × donkey (blue triangle). Equine type explained 23.6% of the total variation in the dataset, and the plot axes are labelled with the amount of variation they represent

RDA using genus level groupings of the OTUs showed that equine type significantly contributed to explaining the observed variation in the fecal anaerobic fungal community composition (*P* < 0.01), and accounted for 23.6% of the overall variation in the dataset. *Caecomyces* was positively associated with pony and pony × donkey. *Piromyces* and the uncultivated genus SK3 were positively associated with pony × donkey and donkey, respectively (Fig. 5). The relative abundance of genera that could not be annotated also seemed to be higher in donkey compared to pony and pony × donkey.

A Kruskal Wallis test showed that only the genus level groups SK3 (*P* = 0.03) and *Piromyces* (*P* = 0.05) were significantly different between equine types. SK3 was detected in only four of the eight donkeys sampled, and none of the animals from the equine types pony × donkey and pony. A Dunn’s Sidak post-hoc analysis indicated that the relative abundance of SK3 was significantly higher in donkey (average percentage relative abundance ± SD; 2.5 ± 3.45) compared to pony × donkey (*P* = 0.03) and pony (*P* = 0.03). In contrast, *Piromyces* was detected in all three equine types. Dunn’s Sidak post-hoc analysis showed that the relative abundance of *Piromyces* was significantly decreased in donkey (0.54 ± 0.88) compared to pony × donkey (3.42 ± 2.68) (*P* = 0.01), with pony (2.13 ± 2.99) not significantly differing from either donkey (*P* = 0.52) or pony × donkey (*P* = 0.26). In pony × donkey, *Piromyces* was detected in all eight animals, whereas in donkey and pony, it was detected in only three and four animals, respectively.

## Discussion

As fecal microbiota is mainly representative of the distal region of the equine hindgut [12], the findings of this study need to be interpreted with this in mind. Fecal microbial concentrations or fecal DM content did not differ between equine types. This new finding suggests that the reported increased fiber degradation in donkeys, compared to ponies, is unlikely to be due to higher concentrations of microbes in the hindgut.

The predominance of the phylum *Firmicutes* in the fecal microbiota of all equines observed in this study is in line with previous reports for horse/pony [23, 26–28, 31, 35–37], donkey [32] and captive Tibetan wild ass [33]. In this study, the fecal prokaryotic community composition was shown to significantly differ between equine types, with donkey being most distinct from both pony and pony × donkey. This novel finding appears to be underpinned by two bacterial genera from the *Lachnospiraceae* family that had a higher relative abundance and prevalence in donkey compared to both pony and pony × donkey: *Lachnoclostridium* 10 and ‘probable genus 10’.

Within the SILVA database version 128, the genus *Lachnoclostridium* 10 contains only one characterized species, *Lachnoclostridium phytofermentans*. The type strain of this species, initially published as *Clostridium phytofermentans* [38], has been effectively (but not validly) published as the type species for the new genus *Lachnoclostridium,* which included a total of 30 different validly described species at the time of its definition in 2013 [39]. *L. phytofermentans* is an obligately anaerobic fibrolytic bacterium that can ferment a wide range of plant polysaccharides [38]. This ability appears to be due to the numerous and diverse range of glycosyl hydrolases encoded within its genome, many of which have been acquired by horizontal gene transfer [40]. Whilst the *L. phytofermentans* type strain was isolated from forest soil, its optimum temperature for growth was reported to be 37 °C with growth observed at pH 6.0–9.0 [38]. One of the other few cultivated members of the genus *Lachnoclostridium* 10 listed in the SILVA database is the rumen bacterium FE2016, which was isolated as part of a study that cultivated plant-attached rumen bacteria (GenBank Accession No. KF698008). As part of the rumen Hungate 1000 project, several ruminal isolates described as belonging to the genus *Lachnoclostridium* have also been genome-sequenced [41].

No characterized species exist within ‘probable genus 10’. However, within the SILVA database this genus does contain 14 cultured bacterial isolates. All of these isolates are of ruminal origin. Three of the isolates were obtained from studies that used modified culturing conditions to isolate novel rumen bacteria: CA43 [42], P18 [43] and NK4A212 [44]. Isolate NK4A212 was proposed as a new genus [44], whilst isolate CA43 was shown to have carboxymethylcellulase and xylanase activity [42]. The remaining 11 isolates are all unpublished, however, ten of them were isolated from the same ‘plant-attached rumen bacteria’ study as the rumen bacterium FE2016.

The isolation of plant-attached rumen bacteria belonging to *Lachnoclostridium* 10 and ‘probable genus 10’, combined with the demonstrated activities of these genera in terms of their ability to degrade polymers present in plant fibers, indicates they are likely to play a role in ruminal fiber degradation. This is likely to also be true of fiber degradation occurring in the equine hindgut. If this is the case, then the higher relative abundances and prevalence of these two genera in donkey compared to pony may contribute to the previously reported increased ability of donkeys, relative to horses/ponies, to digest fiber [8, 9].

Like the prokaryotic community, another novel finding in this study was that the anaerobic fungal community composition was also different between equine types. A higher number of anaerobic fungal OTUs was found in donkey compared to both pony and pony × donkey. As anaerobic fungi can vary in terms of their growth rate, substrate preferences and fiber degrading activity [16, 45], a larger diversity of anaerobic fungi may enable donkeys to utilize fibrous plant material more effectively compared to equines with less diverse anaerobic fungal populations. As anaerobic fungal pure cultures have been reported to have 3–29 different OTUs [46], it is likely that this increased number of OTUs in donkey is representative of one or two extra anaerobic fungal species being present.

Only donkey contained the uncultivated genus SK3 [47, 48], although the genus was only present in half of the animals sampled. Anaerobic fungal sequences belonging to SK3 have been previously obtained from cow manure [49], as well as the rumen of sheep, cattle and deer [48]. SK3 was not previously found in cultivation independent studies of the hindgut digesta of a pony [50] or the fecal material of five different types of domesticated and non-domesticated equines [51]. As such, this appears to be the first report of this genus in equines, and indicates that it is not ruminant specific. SK3 is a sister group to the two bulbous genera *Caecomyces* and *Cyllamyces* [47], suggesting that SK3 may be a third genus that has this type of rhizomycelium.

*Caecomyces* predominated all of the animals in this study, and has previously been reported to occur in the pony caecum and equine feces [51, 52]. The uncultivated genera AL1 (=NG1) and AL3 (=NG3) were previously found to be predominant in the feces of different equine species [51], and AL1 has also been detected along the equine hindgut [50]. However, neither of these genera were detected in this study. Cultivation based studies of anaerobic fungi from domesticated equines have most commonly resulted in the isolation of *Piromyces* [16, 53–55], although other genera have also been reported [52, 56]. In this study, *Piromyces* was detected in all three equine types as a minor genus, but was not detected in all animals. It has been previously shown that a *Piromyces citronii* isolate from a donkey degraded cellulose more rapidly and to a greater extent than a *P. citronii* isolate from a pony [15]. Therefore, whilst no differences in *Piromyces* existed between pony and donkey in this study, it cannot be concluded that the fibrolytic activity of the *Piromyces* in donkey was comparable to that of pony. To this end, studies that assess anaerobic fungal community composition and function at strain-level resolution are warranted.

From this study, the findings clearly indicate that fecal microbial community composition differs between different equine types. When comparing donkey and pony, the observed differences all related to an increase in the relative abundance or diversity of taxa with known, or potential, roles in plant material degradation. These findings are consistent with previous reports of donkeys having an increased ability to digest fiber relative to horses/ponies [8, 9]. As such, this information may provide promising avenues to enhance fiber degradation in the equine hindgut, for example by assessing the probiotic potential of *Lachnoclostridium phytofermentans* or anaerobic fungi to enhance fiber degradation in the equine hindgut.

The driver(s) for the observed difference in fecal microbiota between donkey and pony in this study remains to be determined. For example, the observed differences in the fecal microbiota may be the cause or an effect of the previously reported [8] decreased feed intake and increased MRT in donkeys compared to ponies. Further investigations are therefore now needed to expand on the novel findings reported here, particularly as measurements of intake, digestibility, MRT and fermentation metabolites were not performed in this study alongside the microbiota analysis.

Additional insight may also be gained by studying mules and hinnies separately in future studies. Differences may exist between mules and hinnies due to microbiota acquired from the mother during birth and early life, particularly due to coprophagy [57]. Coprophagic behavior has been speculated to imprint feed selective behavior [58], which also has important implications for the host due to diet having a major impact on the equine hindgut microbiome [5].

## Conclusions

When fed the same forage diet, fecal prokaryotic and anaerobic fungal community composition significantly differed between equine types, but not prokaryotic alpha diversity, fecal microbial concentrations or fecal DM content. Donkeys, compared to both pony and pony × donkey, had higher relative abundances of two bacterial genera, *Lachnoclostridium* 10 and ‘probable genus 10’, that have known, or a potential, role in plant fiber degradation. Furthermore, donkeys also had an increased number of anaerobic fungal OTUs and a higher relative abundance of the uncultivated anaerobic fungal genus SK3 compared with pony and pony × donkey, but a lower relative abundance of *Piromyces* compared with pony × donkey. These findings are consistent with the previously reported increased fiber degradation in donkeys compared to ponies, and suggests that the hindgut microbiota play a role. This offers novel opportunities to generate more energy from dietary fiber through management of the microbiota, decreasing the need for energy dense feeds which are a risk factor for gut-mediated disease.

## Methods

### Animals, diet and management

This study was conducted to assess differences in fecal microbiota between two different equine species (pony, donkey) and their derived hybrids (i.e. pony × donkey). The adult animals (*n* = 8 for each type) used in the experiment were selected for this study based on the following criteria: (i) clinically healthy with no reported medication/illness in the 6 months prior to the study, (ii) no known history of gut-related disorders and (iii) history (health, diet and management) information available for a minimum of 12 months prior to the start of the experiment. Furthermore, the animals involved in this study were all clinically healthy in terms of their parasite profiles, and none of the animals had a history of endoparasite related disease. Further details of the individual animals in terms of their age, sex and weight are given in Additional file 1: Table S1.

During the study, all the animals were group housed in open barns on the same farm, with free access to an outside yard area, water and a mineral lick. For a 4 week period, all animals were provided straw ad libitum and haylage. Haylage was supplied to the group housed animals at amounts known to result in maintenance of consistent bodyweight. All animals were fed exactly the same batches of forage for the last 2 weeks of the 4 week period.

### Fecal sample collection and determination of dry matter content

On the last day of the 4 week period, for each animal the first feces produced after 9 A.M. was collected from the ground immediately following defecation. Parts of the feces that were visibly free of dirt, bedding etc. were placed into a clean bucket and then a pre-weighed tube was filled (approx. 20–30 g wet weight). The filled tubes were then weighed before being placed on wet ice. Samples were kept on wet ice for a maximum of 1 hour before being stored at − 20 °C. Fecal samples were then freeze-dried to a constant weight. For each sample, the percentage fecal dry matter content was then calculated using the original wet weight and the final freeze-dried weight.

### DNA extraction

The freeze dried fecal material was broken up by hand, and any large fibrous particles cut into smaller pieces using a sterile scalpel. The material was then placed into a mortar and manually ground with a pestle. Total DNA was extracted from 25 mg of the freeze-dried and ground fecal samples using the MoBio PowerSoil DNA isolation kit (QIAGEN Benelux BV, Venlo, Netherlands). The manufacturer’s protocol was followed except that after the addition of buffer C1, the samples in the PowerBead tubes were processed in a bead beater (Precellys 24, Bertin technologies, Montigny-le-Bretonneux, France) for 3 × 1 min at 5.5 m/s. DNA extracts were then further purified using the Zymo Research OneStep PCR inhibitor removal kit (BaseClear Lab Products, Leiden, Netherlands) following manufacturer’s instructions. The purity of the resulting DNA extract was assessed using a NanoDrop ND-1000 spectrophotometer (NanoDrop® Technologies, Wilmington, DE, USA), and the quantity determined using a Qubit dsDNA BR assay (Thermo Scientific, Breda, Netherlands).

### Determination of microbial concentrations

For absolute quantification of bacteria and archaea, SYBR green qPCR assays were performed with sample DNA extracts using a CFX384 Touch™ Real-Time PCR Detection System (Bio-Rad Laboratories BV, Veenendaal, Netherlands) as previously described [59]. All qPCR analyses were carried out in triplicate with a reaction volume of 10 μL and 2 ng of sample DNA extracts. Equine specific standard curves (10^8^ to 10^2^ amplicon copies/μL) for the assays were prepared using purified PCR amplicons generated from an equine fecal DNA extract using the primers and cycling conditions previously described for the preparation of qPCR standards [59]. The bacterial PCR amplicons were generated using the primers 27F (5′- AGAGTTTGATCCTGGCTCAG-3′ [60] and PROK1492R (5′- GGWTACCTTGTTACGACTT-3′ [61]). The archaeal PCR amplicons were generated using the primers 25F (5′- CYGGTTGATCCTGCCRG-3′ [62] and PROK1492R (5′- GGWTACCTTGTTACGACTT-3′ [61]).

For absolute quantification of anaerobic fungi, a Taqman probe based method was used as previously described [63] with the exception that a CFX384 Touch™ Real-Time PCR Detection System (Bio-Rad Laboratories BV) was used. All qPCR analyses were carried out in triplicate with a reaction volume of 10 μL, and 20 ng of sample DNA extracts were used. Standard curves (10^8^ to 10^1^ amplicon copies/μL) for the assays were prepared using purified PCR amplicons generated from *Neocallimastix frontalis* strain R_E_1 DNA (kindly provided by Dr. Tony Callaghan, Bavarian State Research Center for Agriculture, Freising, Germany). The PCR amplicon was generated using the primers Neo18SF (5′-AATCCTTCGGATTGGCT-3′ [63] and AF LSU reverse (5′-CTTGTTAAMYRAAAAGTGCATT-3′ [64]).

### Prokaryotic community composition analysis

For 16S rRNA gene based prokaryotic community composition profiling, barcoded amplicons from the V4 region of 16S rRNA genes were generated from the DNA extracts. Primers for the V4 region and individual sample-specific barcoding strategy were as previously described [65]. PCR was performed in a total volume of 50 μL containing 1× HF buffer (Finnzymes, Vantaa, Finland), 1 μL dNTP Mix (10 mM; Promega Benelux, Leiden, Netherlands), 1 U of Phusion® Hot Start II High-Fidelity DNA polymerase (Finnzymes), 500 nM of each sample-specific barcoded primer and 2 ng of sample DNA. The cycling conditions consisted of an initial denaturation at 98 °C for 30 s followed by 25 cycles of 98 °C for 10 s, 56 °C for 10 s and 72 °C for 10 s, and then a final extension at 72 °C for 7 min. Triplicate sample-specific barcoded PCR reactions were prepared for each sample, along with a non-template control (NTC) reaction. The presence of the sample-specific barcoded PCR products was assessed by agarose gel electrophoresis on a 2% (w/v) agarose gel containing 1× SYBR® Safe (Thermo Scientific), and the NTC reactions were confirmed to be negative. Pooled triplicate sample-specific barcoded reactions were then purified using HighPrep™ (MagBio Europe Ltd., Kent, United Kingdom) and quantified using a Qubit dsDNA BR Assay Kit (Thermo Scientific). Purified sample-specific barcoded PCR products were mixed in equimolar amounts into pools together with defined synthetic mock communities which allow assessment of potential technical biases [65]. Pools then underwent adaptor ligation followed by sequencing on the Illumina HiSeq4000 using 150 paired end (PE) sequencing (GATC-Biotech, Konstanz, Germany, now part of Eurofins Genomics Germany GmbH).

The 16S rRNA gene sequencing data was analyzed using NG-Tax 2.0 [66], which executes four major tasks: demultiplexing and amplicon read cleaning, OTU-picking, denoising, and taxonomic assignment. NG-Tax 2.0 defines OTUs using an open reference approach, and OTUs are defined as unique sequences that are above a user-defined minimum abundance threshold. NG-Tax 2.0 was run with the following default settings: 70 nt read length (i.e. 140 nt in total due to being paired-end data), ratio OTU abundance 2.0, classify ratio 0.8, minimum percentage threshold 0.1%, identity level 100% and error correction of one mismatch (98.5%). Paired-end libraries were filtered to contain only read pairs with perfectly matching barcodes, and those barcodes were used to demultiplex reads by sample. The chimera detection process in NG-Tax uses the following condition: if the forward and reverse read of the OTU are identical to two different OTUs in the same sample and the abundance of the matched OTUs are at least twice of the abundance, then the OTU is marked as chimeric. Taxonomy was assigned to OTUs in NG-Tax 2.0 as previously described [66] using the 128 version of the SILVA 16S rRNA gene reference database [67].

### Anaerobic fungal community composition analysis

For anaerobic fungal community composition profiling, barcoded amplicons comprising the partial 18S rRNA gene (~ 130 bp), full ITS1 region and partial 5.8S rRNA gene (~ 31 bp) were generated using a 2-step PCR strategy with a SensoQuest Labcycler as previously described [46]. The first PCR step was performed using previously published ARISA primers [63] with the addition of UniTag adapters (underlined): Neo 18S For 5′-GAGCCGTAGCCAGTCTGCAATCCTTCGGATTGGCT-3′ and Neo 5.8S Rev. 5′-GCCGTGACCGTGACATCGCGAGAACCAAGAGATCCA-3′. PCR was performed in a total volume of 25 μL containing 1× HF buffer, 1 μL dNTP Mix (10 mM), 1 U of Phusion® Hot Start II High-Fidelity DNA polymerase, 500 nM of each primer and 2 ng of sample DNA. The cycling conditions consisted of an initial denaturation at 98 °C for 3 min followed by 40 cycles of 98 °C for 10 s, 58 °C for 30 s and 72 °C for 30 s, and then a final extension at 72 °C for 6 min. Triplicate PCR reactions were prepared for each sample, along with a non-template control (NTC) reaction. The presence of the PCR products was assessed by agarose gel electrophoresis on a 2% (w/v) agarose gel containing 1× SYBR® Safe. Pooled triplicate reactions, as well as the negative NTC reaction (due to the high number of PCR cycles), were then purified using HighPrep™.

The second PCR step was then employed to add an 8 nucleotide sample specific barcode to the 5′- and 3′- end of the PCR products as previously described [59]. Each PCR reaction, with a final volume of 100 μL, contained 5 μL of the purified first step PCR product, 5 μL each of barcoded forward and reverse primers (10 μM), 2 μL dNTP Mix (10 mM), 2 U of Phusion® Hot Start II High-Fidelity DNA polymerase and 1× HF buffer. Amplification consisted of an initial denaturation at 98 °C for 30 s followed by 5 cycles of 98 °C for 10 s, 52 °C for 20 s and 72 °C for 20 s, and then a final extension at 72 °C for 10 min. Barcoded PCR products were then purified using the HighPrep™ and quantified using a Qubit dsDNA BR Assay Kit. Purified barcoded PCR products were then pooled in equimolar amounts along with defined synthetic mock communities [46]. Pools were then sequenced on the Illumina HiSeq 2500 using the Rapid Run 300 bp PE sequencing mode (GATC-Biotech).

The anaerobic fungal sequence data was then analyzed using NG-Tax 2.0 as previously described [46]. NG-Tax 2.0 was run using the default parameters (as specified earlier) except for the following: 150 nt read length (i.e. 300 nt in total due to being paired-end data), minimum percentage threshold 0.6% and error correction of one mismatch (99.3%). As the barcoded amplicon primers used were not within the AF-ITS1 database used for OTU annotation (which is a requirement for annotation by NG-Tax), an empty database file (emptydb.fasta.gz which is available at http://download.systemsbiology.nl/ngtax/databases/) was used and the OTUs then subsequently annotated manually.

Fasta files of the OTUs from the NG-Tax generated biom file were extracted using the script otuseq_export.py (which is available at https://gitlab.com/wurssb/gen_fake_mocks/tree/master/paper_data). The OTUs were annotated using BLASTN searches against the AF-ITS1 database [47] (version 3.3, available from www.anaerobicfungi.org) using default settings with “-num_alignments 10” (BLAST version 2.4.0). For OTUs that could not be annotated by the AF-ITS1 database, BLASTN searches were performed against the NCBI database. Cut-off levels for OTU annotations were determined based on the mean percentage similarities of full-length sequences in the AF-ITS1 database within clade and within genus. These cut-off levels were > 98% for clade and > 95% for genus. The NG-Tax generated biom file was converted to a tab delimited table to enable OTU annotations to be added. The OTUs that were clearly associated with the NTC sample were also manually removed from the tab delimited table at this stage, along with any OTUs that were not anaerobic fungal in origin. The resulting tab delimited table was then converted back to a biom file.

### Statistical analysis

Microbial composition summary box plots and UniFrac based Principal Coordinate Analysis (PCoA) were generated within R (version 3.4.1) [68] using the following libraries and packages: microbiome (https://microbiome.github.io/tutorials/), microbiomeutilities (https://github.com/microsud/microbiomeutilities), RColorBrewer [69], magrittr [70], phyloseq [71], picante [72], nlme [73], vegan [74], lattice [75], permute [76], ape [77], ggplot2 [78], and ggpubr [79]. QIIME 1 [80] was used to generate taxa genus level biom tables using the script “summarize_taxa.py”. Redundancy analysis (RDA) was performed using Canoco 5 [81] to assess the relationship between genus-level phylogenetic groupings of the OTUs and equine type. The QIIME 1 script “group.signficance.py” was used to test differences in relative abundance of individual genera between equine types using Kruskal Wallis with Bonferroni correction of *P* values. A Dunn’s-Sidak post-hoc test with Bonferroni correction was then performed on genera that were significantly different between equine types (*P* < 0.05), in order to determine which equine types significantly differed from each other (MATLAB). The QIIME 1 script “alpha.diversity.py” was used to determine per sample the number of observed OTUs and the Phylogenetic Diversity (i.e. PD_whole_tree) value. Number of observed OTUs, ‘Phylogenetic Diversity’ and fecal dry matter content were analyzed using a one-way ANOVA with equine type as a single independent factor (Genstat 18th Edition, VSN International Ltd.). All qPCR data was analyzed in the same manner after a Log_10_ transformation. For all statistical tests the significance threshold was alpha = 0.05. *P* values for multivariate data were all Bonferroni corrected (as indicated above).

## Supplementary information

**Additional file 1 **: **Table S1.** Details of the animals used in this study.

**Additional file 2 **: **Figure S1.** Boxplot showing the six main bacterial and archaeal phyla detected in the different equine types. The minor phyla (< 1%) are grouped as ‘Other’. Boxes show the 25th and 75th percentiles with the median represented by a horizontal line. Whiskers show the data range with the exception of any outliers, which are indicated as data points.

## Data Availability

The datasets and material supporting the conclusions of this article are provided as follows. Additional information is provided in Additional files 1 and 2. The barcoded amplicon sequence data is deposited in the European Nucleotide Archive under the study accession number PRJEB32772. All the sample barcodes, R codes and data used in the analysis are available at https://github.com/mibwurrepo/EdwardsJ_2019_Equine_Type_Comparison, unless indicated otherwise.

## Abbreviations

AF-ITS1: Anaerobic fungal ITS1
DM: Dry matter
ITS1: Internal Transcribed Spacer 1
MRT: Mean retention time
NTC: Non-template control
OTU: Operational Taxonomic Unit
PCoA: Principal co-ordinate analysis
PCR: Polymerase chain reaction
PE: Paired end
qPCR: Quantitative PCR
RDA: Redundancy analysis
SD: Standard deviation
