# Supplementary material for: Domesticated equine species and their derived hybrids differ in their fecal microbiota
Source: Anim Microbiome. 2020 Mar 16;2:8. doi: 10.1186/s42523-020-00027-7 (PMC7807894; doi:10.1186/s42523-020-00027-7)
Supplement: Supplementary file 1 — Additional file 1 : Table S1. Details of the animals used in this study. [file 42523_2020_27_MOESM1_ESM.docx]

**Table S1:** Details of the animals used in this study.

| **Animal Code** | **Equine Type** | **Sex** | **Age (years)** | **Weight (kg)** |
| --- | --- | --- | --- | --- |
| 1 | Donkey | Gelding | 6 | 145 |
| 2 | Donkey | Female | 19 | 134 |
| 3 | Donkey | Gelding | 15 | 281 |
| 4 | Donkey | Female | 8 | 187 |
| 5 | Donkey | Female | 13 | 162 |
| 6 | Donkey | Gelding | 8 | 210 |
| 7 | Donkey | Gelding | 9 | 230 |
| 8 | Donkey | Gelding | 7 | 214 |
| 9 | Pony | Gelding | 19 | 241 |
| 10 | Pony | Gelding | 21 | 133 |
| 11 | Pony | Gelding | 8 | 126 |
| 12 | Pony | Female | 11 | 215 |
| 13 | Pony | Female | 19 | 403 |
| 14 | Pony | Gelding | 20 | 226 |
| 15 | Pony | Female | 22 | 438 |
| 16 | Pony | Gelding | 14 | 232 |
| 17 | Pony × Donkey | Female | 8 | 236 |
| 18 | Pony × Donkey | Gelding | 4 | 166 |
| 19 | Pony × Donkey | Gelding | 4 | 293 |
| 20 | Pony × Donkey | Gelding | 12 | 258 |
| 21 | Pony × Donkey | Female | 10 | 337 |
| 22 | Pony × Donkey | Female | 5 | 236 |
| 23 | Pony × Donkey | Gelding | 26 | 333 |
| 24 | Pony × Donkey | Gelding | 24 | 180 |
