# Supplementary material for: Domesticated equine species and their derived hybrids differ in their fecal microbiota
Source: Anim Microbiome. 2020 Mar 16;2:8. doi: 10.1186/s42523-020-00027-7 (PMC7807894; doi:10.1186/s42523-020-00027-7)
Supplement: Supplementary file 2 — Additional file 2 : Figure S1. Boxplot showing the six main bacterial and archaeal phyla detected in the different equine types. The minor phyla (< 1%) are grouped as ‘Other’. Boxes show the 25th and 75th percentiles with the median represented by a horizontal line. Whiskers show the data range with the exception of any outliers, which are indicated as data points. [file 42523_2020_27_MOESM2_ESM.pdf]

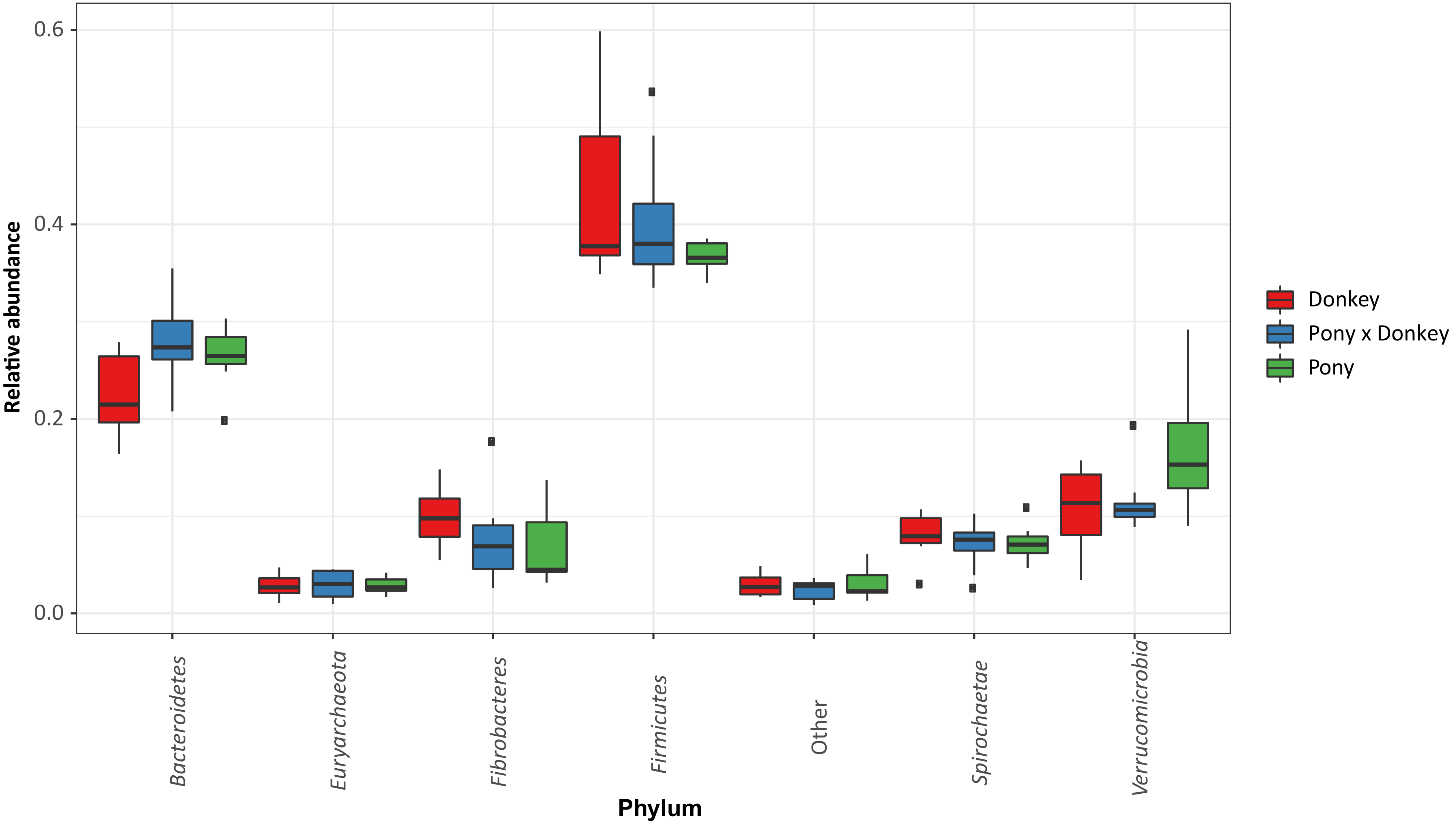

Figure S1. Boxplot showing the six main bacterial and archaeal phyla detected in the different equine types. The minor phyla (<1%) are grouped as 'Other'. Boxes show the 25th and 75th percentiles with the median represented by a horizontal line. Whiskers show the data range with the exception of any outliers, which are indicated as data points.
